# Early administration of umbilical cord blood cells following brief high tidal volume ventilation in preterm sheep: a cautionary tale

**DOI:** 10.1186/s12974-024-03053-3

**Published:** 2024-05-08

**Authors:** Nhi T. Tran, Tayla R. Penny, Kyra YY. Chan, Tanya Tang, Paris C. Papagianis, Tara Sepehrizadeh, Lakshmi Nekkanti, Valerie A. Zahra, Yen Pham, Tamara Yawno, Ilias Nitsos, Sharmony B. Kelly, Alison M. Thiel, Michael de Veer, Dhafer M. Alahmari, Michael C. Fahey, Graham Jenkin, Suzanne L. Miller, Robert Galinsky, Graeme R. Polglase, Courtney A. McDonald

**Affiliations:** 1 The Ritchie Centre, Hudson Institute of Medical Research, Clayton, VIC Australia; 2 Department of Obstetrics and Gynaecology, Monash University, Clayton, VIC Australia; 3 Department of Pharmacology, Biomedicine Discovery Institute, Monash University, Clayton, VIC Australia; 4 Monash Biomedical Imaging, Monash University, Clayton, VIC Australia; 5 Department of Paediatrics, Monash University, Clayton, VIC Australia; 6 Monash Biomedicine Discovery Institute and Department of Medical Imaging and Radiation Sciences, Monash University, Clayton, VIC Australia; 7 Department of Diagnostic Imaging, Kind Saud Medical City, Riyadh, Saudi Arabia

**Keywords:** Umbilical cord blood cells, Preterm brain injury, Ventilation, Neuroinflammation, Stem cell therapy, Preterm ventilation

## Abstract

**Background:**

Umbilical cord blood (UCB) cells are a promising treatment for preterm brain injury. Access to allogeneic sources of UCB cells offer the potential for early administration to optimise their therapeutic capacities. As preterm infants often require ventilatory support, which can contribute to preterm brain injury, we investigated the efficacy of early UCB cell administration following ventilation to reduce white matter inflammation and injury.

**Methods:**

Preterm fetal sheep (0.85 gestation) were randomly allocated to no ventilation (SHAM; *n* = 5) or 15 min *ex utero* high tidal volume ventilation. One hour following ventilation, fetuses were randomly allocated to i.v. administration of saline (VENT; *n* = 7) or allogeneic term-derived UCB cells (24.5 ± 5.0 million cells/kg; VENT + UCB; *n* = 7). Twenty-four hours after ventilation, lambs were delivered for magnetic resonance imaging and post-mortem brain tissue collected. Arterial plasma was collected throughout the experiment for cytokine analyses. To further investigate the results from the in vivo study, mononuclear cells (MNCs) isolated from human UCB were subjected to in vitro cytokine-spiked culture medium (TNFα and/or IFNγ; 10 ng/mL; *n* = 3/group) for 16 h then supernatant and cells collected for protein and mRNA assessments respectively.

**Results:**

In VENT + UCB lambs, systemic IFNγ levels increased and by 24 h, there was white matter neuroglial activation, vascular damage, reduced oligodendrocytes, and increased average, radial and mean diffusivity compared to VENT and SHAM. No evidence of white matter inflammation or injury was present in VENT lambs, except for mRNA downregulation of *OCLN* and *CLDN1* compared to SHAM. In vitro, MNCs subjected to TNFα and/or IFNγ displayed both pro- and anti-inflammatory characteristics indicated by changes in cytokine (IL-18 & IL-10) and growth factor (BDNF & VEGF) gene and protein expression compared to controls.

**Conclusions:**

UCB cells administered early after brief high tidal volume ventilation in preterm fetal sheep causes white matter injury, and the mechanisms underlying these changes are likely dysregulated responses of the UCB cells to the degree of injury/inflammation already present. If immunomodulatory therapies such as UCB cells are to become a therapeutic strategy for preterm brain injury, especially after ventilation, our study suggests that the inflammatory state of the preterm infant should be considered when timing UCB cells administration.

**Supplementary Information:**

The online version contains supplementary material available at 10.1186/s12974-024-03053-3.

## Background

Umbilical cord blood (UCB) cell therapy is emerging as an exciting and highly promising neuroprotective strategy for neonatal morbidities such as preterm brain injury. UCB cells are enriched with multiple stem and progenitor cell types – each possessing synergistic pleiotropic properties that offer cellular and tissue protection [1]. The protective properties of UCB cells are likely to be mediated by the paracrine secretion of cytokines, chemokines and growth factors [2], resulting in immunomodulatory effects [3]. In addition to their immunomodulatory properties, UCB cells have advantages over other cell therapy options as UCB cells are readily available from donors, have a low immunogenicity and greater proliferation profile compared to adult peripheral blood or bone marrow sources of stem cells. As such, multiple clinical trials in preterm infants have already been conducted reporting a good safety profile [4, 5]. In particular, autologous UCB cell (5 × 10^7^ cells/kg) administration in preterm infants ~ 7 h after birth, significantly reduced the required duration of mechanical ventilation (3.2 days vs. 6.41 days) and oxygen therapy (5.33 days vs. 11.31 days) [6]. This is of particular interest as mechanical ventilation and hyperoxia/hypoxia are both independent risk factors for respiratory related preterm brain injury [7, 8]. Moreover, preclinical studies have found that earlier administration of UCB cells at 12 h compared to 5 days after hypoxia-ischemia was more efficacious in reducing white matter injury [9, 10]. This indicates that delaying UCB cells administration also delayed the release of peripheral trophic factors entering the brain parenchyma and thereby diminished their neuroprotective potential. Early UCB cell administration during peak injury response may provide superior brain injury prevention – a key consideration in current clinical and preclinical investigations.

Inflammation is a major underlying pathology and antecedent to preterm brain injury. Inflammatory processes develop over time with characteristic increases in cytokine and chemokine release into the circulation and within the brain, which can have significant downstream effects that lead to brain injury [11]. The key neuroinflammatory pathways that also contribute to the cerebral increases in cytokine and chemokine release is glial activation (microglia and astrocytes) and blood-brain barrier (BBB) breakdown [11, 12]. Collectively, increased neuroinflammatory processes can mediate injury to the immature oligodendrocytes which is thought to underlie the characteristic white matter injury in preterm infants [13]. The exact temporal profile of inflammatory processes is difficult to ascertain within the preterm infant as other confounding underlying pathologies arising *in utero*, perinatally or postnatally, are likely to be involved. Nonetheless, if early UCB cell administration is to be used as a treatment strategy in preterm infants, the effect of UCB cells in a pro-inflammatory environment needs to be investigated.

Respiratory support is a common postnatal clinical intervention in preterm infants where even the brief use of respiratory intervention can trigger inflammatory processes [7, 14]. Indeed, within 2 h of protective mechanical ventilation of human preterm infants, levels of multiple pro-inflammatory cytokines (IL-8, IL-1β, TNF) in arterial blood are increased [15], which persists with prolonged durations of ventilation [16]. We have previously demonstrated in preterm lambs that there is peak upregulation of pro-inflammatory cytokine transcripts (*IL1B*, *IL6*) levels in the brain and activation of glial cells within the cerebral white matter ~ 2 h after high tidal volume ventilation (V_T_; targeting 10–15 mL/kg for 15 min) [7, 17, 18]. Administration of UCB cells during this peak inflammatory response may blunt the systemic inflammatory response and ultimately, protect the brain from injury.

Here, we first utilised the fetal head-out ventilation technique in preterm fetal sheep at 125 days of gestation (dGA) [14] to investigate the effects of early UCB cells administration on systemic inflammatory cytokine levels and cerebral white matter 24 h following brief high tidal volume (V_T_) injurious ventilation. High V_T_ ventilation was utilised as the injury model as more than 85% of preterm infants inadvertently receive high V_T_ in the delivery room [19], which has been associated with haemorrhagic and diffuse white matter brain injury [20–22]. As we have shown that a peak inflammatory response occurs < 2 h after 15 min of high V_T_ ventilation [7, 17, 18], this study aims to assess whether UCB cells treatment at 1 h after 15 min of high V_T_ ventilation is able to attenuate this peak inflammatory response and reduce overall white matter injury. To further characterise how UCB cells respond to pro-inflammatory environments, we isolated mononuclear cells (MNCs) from human UCB in vitro and exposed the cells to pro-inflammatory cytokines. The cells and supernatant were analysed for inflammatory markers and growth factors to assess the influence of an inflammatory environment on the functional characteristics of the UCB cells. We hypothesised that in vivo administration of UCB cells during the early inflammatory period 1 h following brief high V_T_ ventilation would mitigate the upregulation of inflammation and subsequent white matter injury in preterm sheep. We also hypothesised that the MNCs contained within UCB would be modulated according to their environment in vitro and a highly inflamed environment may skew the function by altering their expression of chemokine-related receptors and release of pro/anti-inflammatory cytokines and growth factors.

## Materials and methods

### Study design

The first study was an in vivo investigation of UCB administration at a time of heightened systemic inflammation induced by brief high V_T_ injurious ventilation in an established fetal ovine model to assess the effects on white matter inflammation and injury. Animal experimental procedures were approved by the Monash Medical Centre Animal Ethics Committee A, Monash University (MMCA/2018/05). All experiments were conducted in accordance with guidelines established by the National Health and Medical Research Council of Australia. All experiments were performed in accordance with the ARRIVE guidelines 2.0 (Supplementary Data File 1) [23].

We then conducted an in vitro study to investigate the characteristic alterations to cells isolated from human-derived UCB following exposure to an inflammatory environment induced by cytokine-spiked media. All human-derived samples were collected with human ethics approval from Monash Health Human Ethics Committee (12387B). Written informed consent was obtained from all participants prior to collection of UCB.

### In vivo sheep preparation

The use of these animals and relevant methodology have previously been published [24, 25] and experimental protocols unique to this study are reported in detail below. Prior to surgery, pregnant ewes at 125 ± 1 day gestation (term ~ 148 days) were randomly assigned to a SHAM (*n* = 5), VENT (*n* = 7) or VENT + UCB (*n* = 7) group. Male to female ratio for fetuses within each group was SHAM, 3:2; VENT, 5:2; and VENT + UCB, 4:3. As previously described [24, 25], sterile surgery was performed with a midline laparotomy on pregnant ewes to expose the fetus head and neck for instrumentation of polyvinyl catheters in the left carotid artery (for arterial blood sampling) and jugular vein (for UCB administration), and an ultrasonic flow probe (3PS; Transonic Systems, NY, USA) around the right carotid artery to measure carotid blood flow (CBF). To allow ventilation, the fetus’ chest was exteriorised and the fetus was intubated with a cuffed endotracheal tube (ID 4.0-4.5 mm; Smiths Medical, UK) and lung liquid passively drained.

Ventilation for the VENT and VENT + UCB groups was conducted under sterile conditions using a neonatal positive pressure ventilator (Babylog 8000+, Dräger, Lübeck, Germany) as described previously [24, 25]. Briefly, the fetus was ventilated for 15 min with a high tidal volume (V_T_) strategy targeting 12–15 mL/kg (normal V_T_: 5–7 mL/kg). SHAM animals remained intubated and exteriorised for 15 min without mechanical ventilation. CBF and V_T_ were continuously measured for the 15 min and digitised using a PowerLab A-D converter and stored on disk using LabChart7 software (AD Instruments, Bella Vista, NSW, Australia). CBF could not be recorded from one lamb from the VENT group due to technical error. Arterial blood gas was measured at 0 (pre-ventilation), 5, 10 and 15 min of ventilation (ABL80 FLEX, Radiometer Medical ApS, Denmark).

After 15 min of ventilation, the carotid flow probe was removed, and fetuses were extubated and returned to the uterus. The fetal jugular vein and carotid artery catheters were externalised through the ewe’s right flank. All incision sites were sutured closed and the ewe and fetus allowed to recover. Analgesia (buprenorphine; 0.3 mg i.m.; Temgesic; Reckitt Benckiser, UK and transdermal fentanyl patch; 75 µg/h; Janssen-Cilag, NSW, Australia) was administered for post-surgery pain relief. Arterial blood gas measurements and arterial samples were collected in heparinised tubes regularly throughout the 24 h study period.

### UCB cells treatment

UCB cells were obtained from healthy term lambs (141 days gestation of term 148 days) undergoing procedures in a separate study, using UCB collection, storage and preparation protocols as previously described [9, 26]. On the day of cell administration, UCB cell samples were rapidly thawed, washed and resuspended in media (Gibco, USA), and cell number and viability measured (minimum 75% viability accepted). At 1 h post-ventilation, ~ 80 million allogeneic UCB cells suspended in 3 mL of phosphate-buffered saline (PBS; Gibco, MA, USA) were administered to VENT + UCB fetuses via the jugular vein catheter, which was then flushed with sterile heparinised saline to ensure all cells were administered [25]. This dosage and administration regime was based on previous studies reporting reduced cerebral inflammation, white matter injury, oxidative stress and cell death after hypoxia-ischemia in fetal sheep [9].

### Magnetic Resonance Imaging (MRI) and post-mortem brain collection

Twenty-four hours post-*in utero* ventilation, ewes were anaesthetised and the fetus exteriorised via the same incision site. All lambs were dried, intubated, and stabilised with a standard protective ventilation strategy (Babylog 8000+, Dräger) using volume guarantee set at 7 mL/kg and PEEP of 5 cmH_2_O. Once the lambs were stabilized, the umbilical cord was clamped and the lambs delivered. Surfactant was administered (100 mg/kg, Curosurf^R^, Chiesi Pharma, Italy) and mechanical ventilation was provided targeting 5 mL/kg. Lambs were then transferred to the 3T MRI system (Siemens Skyra, Erlangen, Germany) at Monash Biomedical Imaging (Clayton, Australia), with a 15-channel radio frequency transmitter/receiver knee coil. Lambs were placed in supine position and ventilation maintained using a BabyPAC portable and MR-compatible ventilator (Pneupac, Smiths Medical, UK). Due to technical issues, MRI results from one lamb from the VENT group and two lambs from the VENT + UCB group were not suitable for analysis; thus the revised group numbers for this analysis were VENT, *n* = 6; VENT + UCB, *n* = 5. MRI acquisition protocols for T2-weighted image and Diffusion Weighted Imaging (DWI) were conducted as previously published [24, 27, 28]. Total acquisition time was around 40 min. Lambs were then euthanised (sodium pentobarbitone > 100 mg/kg i.v.; Valabarb Euthanasia Solution; Jurox, NSW, Australia) immediately after scanning was completed. Ewes were similarly euthanized immediately after delivery of the lambs.

At post-mortem, the lamb’s brain was removed and the cerebrum hemisected along the medial longitudinal fissure. The periventricular and subcortical white matter (PVWM and SCWM respectively) of the left cerebral hemisphere were dissected and snap-frozen in liquid nitrogen. The right cerebral hemisphere was cut coronally into 5 mm blocks and immersion fixed in 10% neutral buffered formalin (Amber Scientific, WA, Australia) for paraffin-embedding.

### MRI data analysis

All data were analysed using the FMRIB Software Library (FSL, FMRIB, Oxford, UK [29]). Pre-processing steps started with extracting brains and creating brain mask using Brain Extraction Tool. All masks were manually brushed and cleaned thoroughly to remove skull and areas other than brain. All DWI data were then corrected for eddy-current distortion using eddy, and then DTI parametric maps of fractional anisotropy (FA), axial diffusivity (AD), radial diffusivity (RD), and mean diffusivity (MD) created with the Diffusion Toolbox of FMRIB. RD is the mean of the second and third eigenvalues (L2 and L3). Region of interests (ROI) were defined on high resolution T2 images to maximise accuracy. Selected ROI: the periventricular white matter (PVWM), the frontal white matter (FWM) was identified using The Sheep Brain Atlas from Michigan State University (Brain Biodiversity Bank, National Science Foundation). High-resolution T2 images of the brains along with the ROIs were co-registered to DWI images using the Linear Image Registration Tool (FLIRT [29]) as previously published [24, 27, 28]. The mean value from 10 adjacent slices within the given ROI volume was calculated; within-animal ROI variability was < 16.8%. MRI examination, image processing and analysis of the structural MR images were performed by persons blinded to group allocations.

### Immunohistochemistry and quantitative analysis

Coronal sections (8 μm) at the level of the frontal, parietal, temporal, and occipital lobes (4 slides/animal/antibody) were stained with rabbit anti-ionised calcium binding adapter molecule-1 (Iba-1; 1:1500, Wako Pure Chemical Industries, Osaka, Japan) to identify microglia; rabbit anti-sheep serum (1:1000, Sigma-Aldrich, USA) to identify vascular extravasation of protein; mouse anti-oligodendrocyte transcription factor-2 (Olig2; 1:1000, Merck, Darmstadt, Germany) for oligodendrocytes; rat anti-myelin basic protein (MBP; 1:200; Merck, Darmstadt, Germany) for mature myelin and oligodendrocytes; and rabbit anti-glial fibrillary acidic protein (GFAP; 1:400, Sigma-Aldrich, USA) to identify astrocytes as described previously [30]. Briefly, sections were pre-treated with citrate buffer (pH 6.0) in a microwave oven. Sections were incubated with secondary biotinylated IgG antibody raised against the corresponding animal secondary (1:200; Vector Laboratories, CA, USA) and reacted using the Vectastain Elite ABC Kit (Vector Laboratories, CA, USA). A ‘Terminal deoxynucleotidyl transferase dUTP nick end labelling’ (TUNEL) assay (ApopTag® Peroxidase In Situ Apoptosis Detection Kit, Millipore, USA) was also conducted according to manufacturer’s instructions. Negative controls (primary antibody omitted) demonstrated no positive staining.

Analyses were conducted at equivalent sites within the PVWM and SCWM of sections from the frontal, parietal, temporal, and occipital lobes of each lamb. Slides were coded and the assessors blinded to the groups. Analysis was conducted using either ImageScope (Aperio Technologies; Leica Biosystems, Germany) or ImageJ software (National Institutes of Health, USA) as described previously [30]. Cell density (cells/mm^2^) of Iba-1^+^ microglia (resting, ameboid and total), GFAP^+^ astrocytes, TUNEL^+^ cells, Olig2^+^ oligodendrocytes, MBP^+^ cells and number of blood vessel profiles with protein extravasation were manually quantified in 3 non-overlapping fields in the PVWM and 6 non-overlapping fields in the SCWM from 2 separate gyri in each section (area = 0.14 mm^2^). The fractional area coverage (%) of Iba-1, GFAP and MBP immunoreactivity was assessed within the same fields. For Iba-1 immunostaining, the total number of microglial aggregations and the fractional area coverage of these aggregations within the PVWM and SCWM were also assessed. Data from all fields were averaged for each brain region then averaged across subjects in each experimental group.

### Magpix cytokine analysis

Arterial plasma was collected following ventilation (0 [pre-ventilation], 15 min [end of ventilation] and 1, 3, 6, 12 and 24 h [post-ventilation recovery]) and used to quantify levels of IFNγ, IL-1β, IL-10, TNFα, IL-8 and IL-6 using a Milliplex MAP bovine cytokine magnetic bead panel assay kits according to manufacturer’s instructions (cat#: CYT1-91 K; MerckMillipore, USA). In brief, 96-well plates were coated with samples, assay buffer, serum matrix and antibody-immobilised beads. Plates were incubated overnight at 4 °C, washed and incubated with detection antibodies for 1 h. Streptavidin-phycoerythrin was added to the plates for 30 min. Sheath fluid was added to the plates and cytokine concentrations were quantified using a Bio-Plex MAGPIX® Multiplex reader with xPOTENT® software (Bio-Rad, CA, USA).

### In vitro UCB mononuclear cell isolation

For in vitro studies, human term UCB samples were obtained from women with uncomplicated pregnancies undergoing elective caesarean section at term (> 37 weeks gestation). Women gave written, informed consent for the collection of their UCB. After clamping of the cord and delivery of the placenta, UCB was collected from the umbilical vein using blood collection bags containing anticoagulant (Macopharma, Tourcoing, France). On average, ~ 100 mL of UCB was collected. UCB was stored at room temperature for up to 48 h until processing and cell isolation. To obtain the mononuclear layer of cells, UCB was transferred to 50 mL falcon tubes and diluted 1:1 with PBS. This was centrifuged at 3100 rpm for 12 min, with no brake. The mononuclear cells were separated and washed in 20 mL PBS and centrifuged at 800 *g* for 5 min to isolate a cell pellet. Red blood cell lysis buffer (ammonium chloride, potassium bicarbonate and EDTA dissolved in double distilled water; Sigma-Aldrich, USA) was added for 3–5 min to lyse excess red blood cells. The reaction was stopped with excess media (16.5% fetal bovine serum and DMEM/F12; Gibco, USA), followed by centrifugation at 400 *g* for 5 min and the supernatant was then aspirated. Cell viability was determined using trypan blue exclusion dye (Gibco), and cells were counted with a hemocytometer. The mononuclear cells were then either used for magnetic bead separation of individual cell types or cryopreserved for later use. For cryopreservation, UCB mononuclear cells were frozen at a density of 20 × 10^6^ cells/mL; in 40% complete media (DMEM/F12, 16.5% FBS, 1% antibiotics), 50% fetal bovine serum (FBS; Gibco) and 10% dimethyl sulfoxide (DMSO; Sigma Aldrich). Cells were then transferred to freezer tubes and left in a freezing container (MrFrosty, Thermo Fisher Scientific) overnight at -80°C, following which they were transferred to liquid nitrogen. To thaw, sample tubes were quickly removed from liquid nitrogen and placed directly into a 37°C water-bath until thawed. Samples were washed to remove DMSO, and cell counts and viability were determined.

### In vitro stimulation of cells

For stimulation of cells, three separate donors were used for each cell type and each stimulation experiment was performed in triplicate. Briefly, cells were rapidly thawed and washed with media (DMEM/F12, 10% FBS and 1% Antibiotic-Antimycotic, Gibco), then centrifuged at 480 *g* for 10 min, the supernatant was aspirated and cells were resuspended in media. Cells were counted using a hemocytometer and viability assessed using trypan blue exclusion dye.

There were four conditions: media alone, media with TNFα, media with IFNγ, and media with TNFα & IFNγ combined. 48-well plates were set up, the four conditions were plated in triplicate for supernatant collection. For RNA, the four conditions were plated in duplicate. After the cells were initially seeded, the plates were incubated at 37°C with 21% O_2_ and 5% CO_2_ overnight to allow the cells to recover from thawing after cryopreservation. After 24 h of recovery, supernatant was removed and the cells were exposed to media only, 10 ng/mL of TNFα, 10 ng/mL of IFNγ or both 10 ng/mL of TNFα and 10 ng/mL of IFNγ. Media was added to increase the final volume in the wells to 500 uL. The plates were incubated for 16 h at 37°C with 21% O_2_ and 5% CO_2_. Supernatant was collected for protein analysis and cells were harvested for either flow cytometry or RNA isolation.

### Luminex protein analysis

Neat cell supernatant was placed into 96-well plates and analyzed with a Magnetic Luminex protein array system (Human Premixed Multi-Analyte Kit; RD Systems, Minneapolis, MN, USA) for the presence of Angiopoietin-1 (Angpt1), SDF-1, IFN-γ, IL-6, VEGFA, BDNF, GDNF, IL-10 and TNFα. This assay is designed for the simultaneous detection of multiple human biomarkers in cell culture supernatants and the assay was performed strictly according to the manufacturer’s instruction (Catalogue Number LXSAHM).

### *mRNA* expression

mRNA expression of genes of interest from frozen tissue are outlined in Table S1. RNA extraction, cDNA preparation and analysis were conducted as described previously [31]. Briefly, mRNA was extracted from the PVWM and SCWM (RNeasy Midi RNA Extraction kit; Qiagen, VIC, Australia) and reverse-transcribed into cDNA according to the manufacturer’s instructions (SuperScript® III First-Strand Synthesis System for RT-qPCR kit; Invitrogen). Genes of interest were measured by quantitative PCR using the Fluidigm Biomark HD system (Fluidigm Corporation, CA, USA). Samples were run in triplicates, averaged and normalised to endogenous housekeeping gene ribosomal protein S18 (RPS18) expression then expressed relative to SHAM group using the 2^–∆∆Ct^ method.

mRNA expression within cultured cells of genes of interest is outlined in Table S2. Cells were harvested for RNA isolation after the 16 h of stimulation. In a modification of the protocol included in the Bioline RNA kit, the cells were first centrifuged after collection, the supernatant was aspirated off and the cells were suspended in 2 µL TCEP and 100 µL RNA Lysis Buffer RL. This solution was then frozen at -80°C until ready for use. After thawing, RNA was isolated according to manufacturer’s instructions (Bioline, Meridian Bioscience, USA). The concentration of the isolated RNA was measured on a Nanophotometer (Implen). RNA was reverse-transcribed into cDNA (SuperScript III reverse transcriptase, Invitrogen; Life Technologies, USA) according to manufacturer’s instructions. Relative mRNA expression was measured by quantitative real-time PCR using Applied Biosystems 7900HT Fast Real-Time PCR system. The expression of all genes was normalized to β-actin for each sample using the 2^–∆∆Ct^ method.

### Statistical analysis

Statistical analyses were conducted using GraphPad Prism (version 10.1.1; GraphPad Software, CA, USA). Data were tested for normality by Shapiro-Wilk test and statistical analysis conducted accordingly. Data violating the assumption of sphericity were corrected with the Greenhouse-Geisser method. An alpha of *P* < 0.05 was adopted to establish statistical significance. All data (Flow cytometry, DTI parameters, proteins, RT-qPCR and immunohistochemistry analyses) were compared using a one-way ANOVA, and significance followed by post-hoc testing using Tukey’s multiple comparisons. For non-parametric data, Kruskal-Wallis test was performed and a Dunn’s post-hoc analysis used. Blood gas, CBF and ELISA data were compared using two-way repeated measures ANOVA. The independent variables assessed were group (*P*_GROUP_) and time of measurement (*P*_TIME_). Where there was a significant interaction between independent variables (*P*_GROUP X TIME_), a post-hoc analysis with Tukey’s multiple comparisons test was undertaken. DTI data are presented as box plots with 5–95% confidence intervals of median, with maximum–minimum error bars. All other data are presented as mean ± SD.

## Results

### Head-out ventilation in preterm fetal sheep and UCB cells administration

**Fig. 1:**
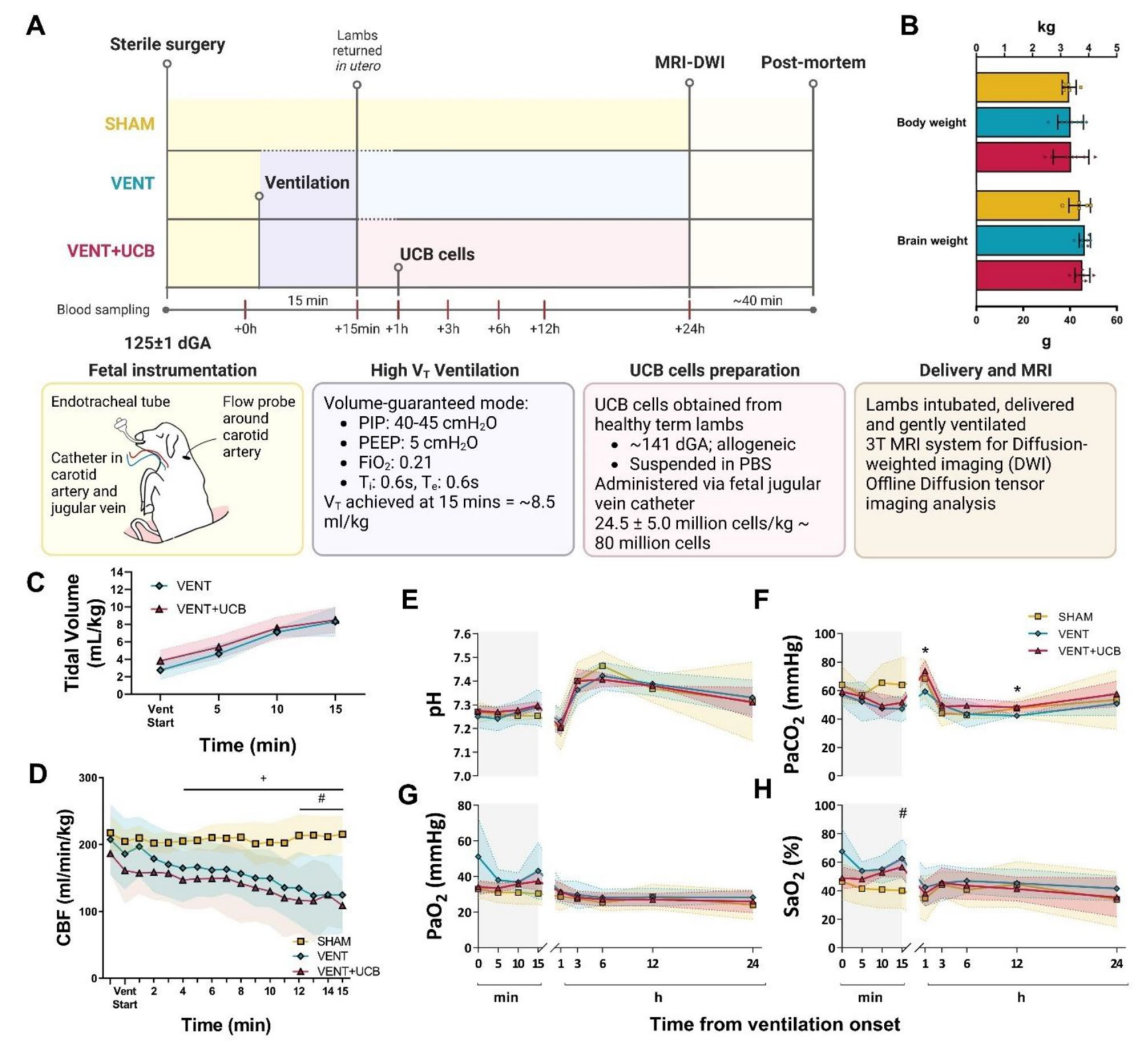
Head-out ventilation in preterm fetal sheep and UCB cells administration. (**A**) Schematic overview of in vivo experimental paradigm in fetal sheep at 125 ± 1 dGA. All fetuses underwent sterile instrumentation. VENT and VENT + UCB fetuses underwent 15 min of high tidal volume (V_T_) ventilation. All fetuses were then returned *in utero* for 24 h of recovery. VENT + UCB fetuses were administered umbilical cord blood (UCB) cells 1 h post-ventilation. All fetuses were delivered at 24 h for MRI. (**B**) Body weight and brain weight were not different between groups. (**C**) V_T_ increased over time in VENT and VENT + UCB fetuses to the same degree. SHAM; *n* = 5, VENT; *n* = 7 and VENT + UCB; *n* = 7. (**D**) Carotid blood flow (CBF) decreased during 15 min of high V_T_ ventilation. Two-Way repeated measures ANOVA. ^#^*P* < 0.05 SHAM vs. VENT; ^+^*P* < 0.05 SHAM vs. VENT + UCB. SHAM; *n* = 5, VENT; *n* = 6 and VENT + UCB; *n* = 7. (**E-H**) Arterial blood gas parameters during 15 min of ventilation (grey) and recovery period. (**E**) pH, (**F**) arterial partial pressure of carbon dioxide (PaCO_2_), (**G**) arterial partial pressure of oxygen (PaO_2_), and (**H**) arterial oxygen saturation level (SaO_2_) at timepoints relative to ventilation. Two-Way repeated measures ANOVA. ^#^*P* < 0.05 SHAM vs. VENT; *P < 0.05 VENT vs. VENT + UCB. SHAM; *n* = 5, VENT; *n* = 7 and VENT + UCB; *n* = 7. All data in (**B-H**) are mean ± SD

Singleton fetuses at 125 ± 1 days of gestational age (dGA) from all three groups had similar body and brain weights (Fig. 1A, B). Ventilation with an intact umbilical cord achieved a V_T_ of ~ 8.40 mL/kg by the end of the 15 min ventilation strategy in all ventilated fetuses, with no difference in delivered V_T_ between groups (VENT: 8.32 ± 1.67; VENT + UCB: 7.77 ± 2.36 mL/kg; *P* = 0.624) (Fig. 1C). Higher V_T_ could not be achieved in line with the low compliance and high airway resistance of the immature lungs characteristic of preterm infants [32]. Carotid blood flow (CBF) gradually decreased during the high V_T_ ventilation in both ventilation groups dropping to 40–42% of baseline by 15 min (Fig. 1D). Fetal pH, PaCO_2_, and PaO_2_ were not altered during high V_T_ ventilation (Fig. 1E-H). At 15 min of ventilation compared to the SHAM group, SaO_2_ was significantly higher in the VENT group and trended higher in the VENT + UCB group (*P* = 0.035 and 0.077 respectively; Fig. 1H).

To test our hypothesis that early UCB treatment would attenuate the peak upregulation of cerebral pro-inflammatory mediators that occurs after > 1 h following high V_T_ ventilation [7, 17, 18], UCB cells administration was conducted 1 h post-ventilation – a clinically feasible timing for an intervention. One hour after the initiation of ventilation, VENT + UCB fetuses were administered allogeneic 24.5 ± 5.0 million UCB cells/kg (~ 80 million cells) intravenously obtained from healthy term lambs. UCB administration did not alter pH, PaO_2_ and SaO_2_ during the 24 h recovery period, but VENT + UCB fetuses had a higher PaCO_2_ levels compared to the VENT group at 1 and 12 h (Fig. 1D-G).

### UCB cell administration following brief high V_T_ ventilation induces white matter injury

**Fig. 2:**
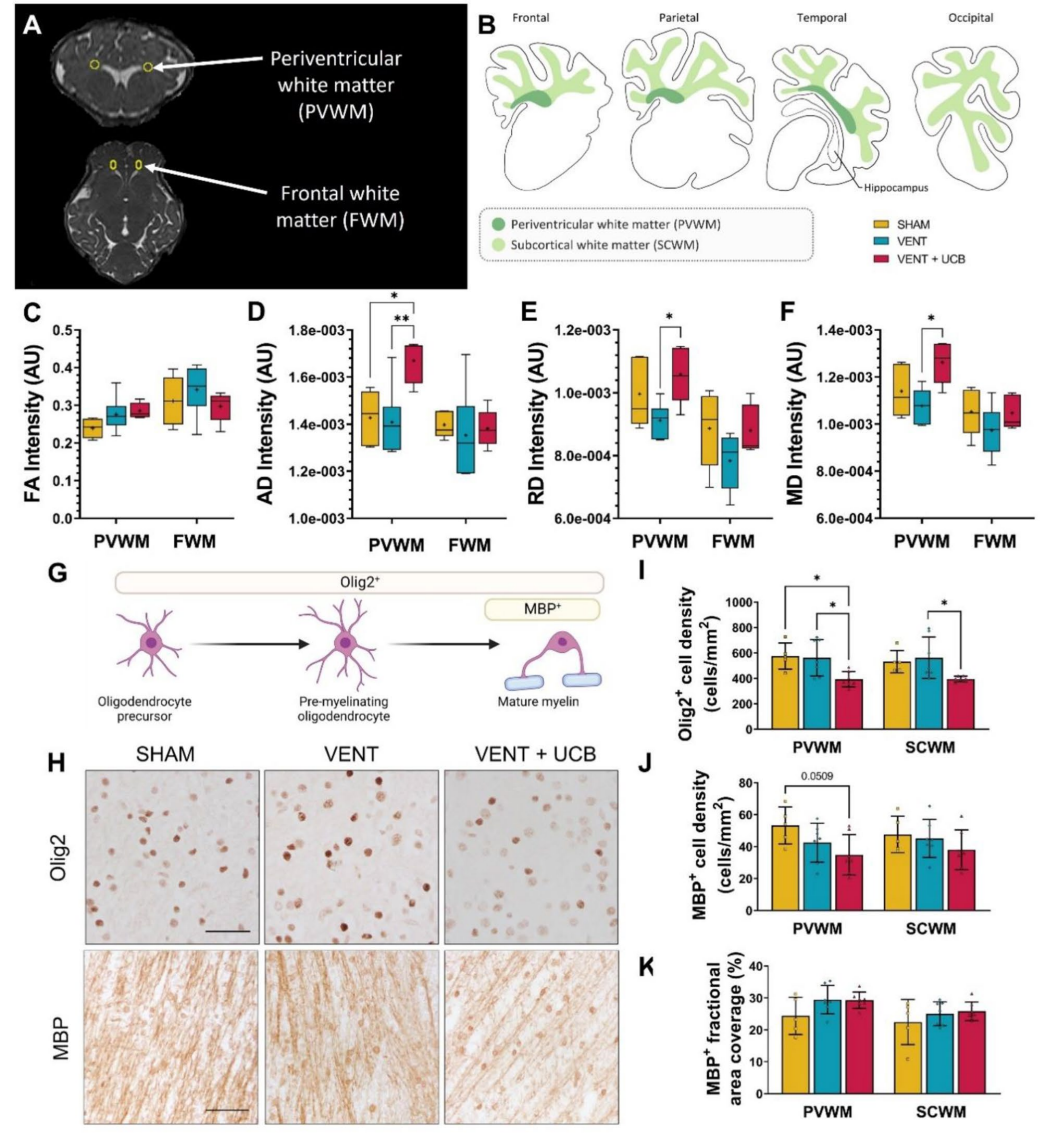
White matter disturbances present in VENT + UCB lambs but not VENT only lambs. (**A**) Representative MRI images of ROIs (yellow circles) in the periventricular white matter (PVWM) and frontal white matter (FWM) for DTI analysis. Figure is reproduced from [28]. (**B**) Immunohistochemical analyses conducted in the PVWM and subcortical white matter (SCWM) across the major cerebral lobes (frontal, parietal, temporal and occipital). (**C-F**) MRI-DTI analysis of **C**) mean fractional anisotropy (FA), (**D**) axial diffusivity (AD), (**E**) radial diffusivity (RD), and (**F**) mean diffusivity (MD) measurements; units = arbitrary unit (AU). Box plots showing interquartile range and min-max, the line marks the median and + marks the mean; SHAM; *n* = 5, VENT; *n* = 6 and VENT + UCB; *n* = 5. One-Way ANOVA, Tukey’s post-hoc comparisons; *P < 0.05, **P < 0.01. (**G**) Olig2^+^ immunostaining marks all oligodendrocyte lineage while MBP + immunostaining marks mature myelin. (**H**) Representative images of Olig2 and MBP immunoreactivity in the PVWM. Scale bar = 50 μm. Quantitative analysis of (**I**) Olig2^+^ and (**J**) MBP^+^ cellular population (cell density) and (**K**) MBP^+^ fractional area coverage. One-Way ANOVA, Tukey’s post-hoc comparisons. All data (**I-K**) are mean ± SD and SHAM; *n* = 5, VENT; *n* = 7 and VENT + UCB; *n* = 7 and *P < 0.05, **P < 0.01

The effects of brief high V_T_ ventilation and UCB cells on white matter were assessed using magnetic resonance imaging-diffusion tensor imaging (MRI-DTI) (Fig. 2A) and immunohistochemical staining (Fig. 2B) in multiple white matter regions of interest.

**Fig. 3:**
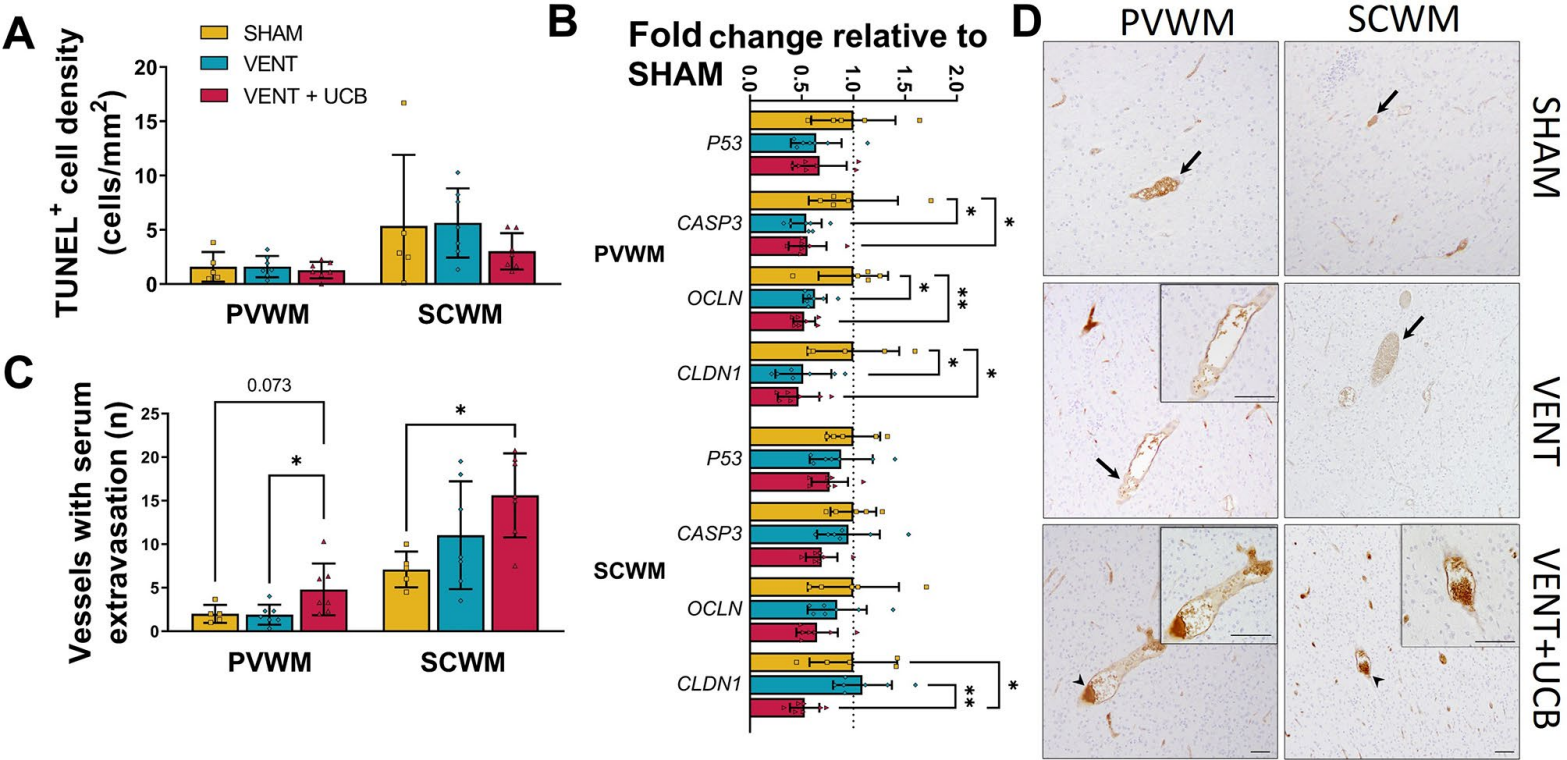
Increased vasculature damage within white matter in VENT + UCB group. (**A**) Quantitative analysis of TUNEL^+^ cell population (cell density) in the PVWM and SCWM. One-Way ANOVA. SHAM; *n* = 5, VENT; *n* = 7 and VENT + UCB; *n* = 7 (**B**) Gene expression levels of cell death related genes (P53 [*P53*] and caspase3 [*CASP3*]) and tight junction related genes (occludin [*OCLN*] and claudin1 [*CLDN1*]) expressed as a fold change relative to SHAM. SHAM; *n* = 5, VENT; *n* = 7 and VENT + UCB; *n* = 7. One-way ANOVA. Tukey’s post-hoc comparisons; *P < 0.05, **P < 0.01. (**C**) Quantitative analysis of the number of vessels with serum extravasation. SHAM; *n* = 5, VENT; *n* = 7 and VENT + UCB; *n* = 7. One-Way ANOVA, Tukey’s post-hoc comparisons; *P < 0.05. All data are mean ± SD. (**D**) Representative images of sheep serum-positive staining in the PVWM and SCWM (arrow indicates intact vessel, arrowhead indicates vessel profile with protein extravasation). Scale bar = 50 μm

In the periventricular white matter (PVWM), mean fractional anisotropy was not different between groups (Fig. 3C). For measurements of axial, radial and mean diffusivity within the PVWM, brief high V_T_ ventilation alone did not result in changes to DTI measurements compared to SHAM, however the administration of UCB cells significantly increased axial, radial and mean diffusivity in the VENT + UCB group compared to VENT (*P* = 0.009, 0.037, 0.013 respectively) (Fig. 3D-F). The increases in axial, radial and mean diffusivity in the VENT + UCB group suggests a degree of alteration to the structures within the major white matter tract, demyelination or increased edema [33]. All mean DTI measurements were similar across groups within the frontal white matter (FWM) (Fig. 3C-F).

White matter was assessed using immunostaining of Olig2^+^, a marker for all oligodendrocyte lineage cells, and MBP^+^, a marker for mature myelin, in the PVWM and subcortical white matter (SCWM) (Fig. 2B,G&H). Ventilation alone did not alter the white matter oligodendrocyte cell density compared to SHAM. The administration of UCB cells however significantly reduced oligodendrocyte number in the PVWM and SCWM in the VENT + UCB group compared to VENT (*P* = 0.026 and 0.030 respectively; Fig. 2I). Assessment of mature myelin (MBP; Fig. 3G&H) revealed no changes in cell density or fractional area coverage within the PVWM and SCWM; though there was a near-significant decrease in the number of MBP^+^ cells in the PVWM in the VENT + UCB group compared to SHAM (*P* = 0.051; Fig. 2J&K). Together, the administration of UCB cells following brief high V_T_ ventilation reduced the number of oligodendrocytes, with a trending decrease in axonal myelination.

This loss of oligodendrocyte populations within the white matter was however not due to apoptosis as indicated by the lack of increased TUNEL^+^ staining within the same areas (Fig. 3A). A lack of apoptosis is consistent with the decreased mRNA levels of *CASP3*, the gene that encodes caspase3, in the VENT + UCB group compared to SHAM (*P* = 0.007; Fig. 3B). Moreover, the decrease in *CASP3* mRNA also occurred in the VENT group when compared to SHAM (*P* = 0.001; Fig. 3B). This indicates that the downregulation of caspase3 activity is a result of the brief high V_T_ ventilation within the white matter tract and is not mediated by UCB cells.

Blood vessel integrity was assessed by counting the number of blood vessels with serum extravasation. VENT + UCB had a greater number of vessels with serum extravasation compared to SHAM in the SCWM (*P* = 0.024; Fig. 3C&D). In the PVWM, the number of vessels with serum extravasation was significantly higher in the VENT + UCB compared to VENT group, and a trend for a significant difference compared to SHAM (*P* = 0.040 and 0.073 respectively; Fig. 3C&D). Increased blood vessel extravasation suggests blood-brain-barrier (BBB) disruption in the VENT + UCB group, which is consistent with the decreased mRNA levels of *OCLN* and *CLDN1*, key tight junction proteins, in the PVWM and SCWM compared to the SHAM group (Fig. 3B). Interesting to note is that *OCLN* and *CLDN1* mRNA levels in the PVWM were also decreased in the VENT group compared to SHAM (*P* = 0.012 and 0.038 respectively; Fig. 3B). At the transcriptional level, the downregulation of *OCLN* enhances intracellular free Ca^2+^ concentration thereby increasing cellular permeability [34]. Claudin-1 has a complex BBB destabilising role and its downregulation contributes to BBB leak [35]. The data suggest that while brief high V_T_ ventilation alone is not causing overt white matter changes at 24 h, there is indication of cellular dysregulation likely involving the integrity of the BBB. The early administration of UCB cells following brief high V_T_ ventilation disturbed white matter integrity, caused a reduction in the oligodendrocyte population and increased permeability of the BBB.

### UCB cells following brief high V_T_ ventilation induces systemic and cerebral inflammation

**Fig. 4:**
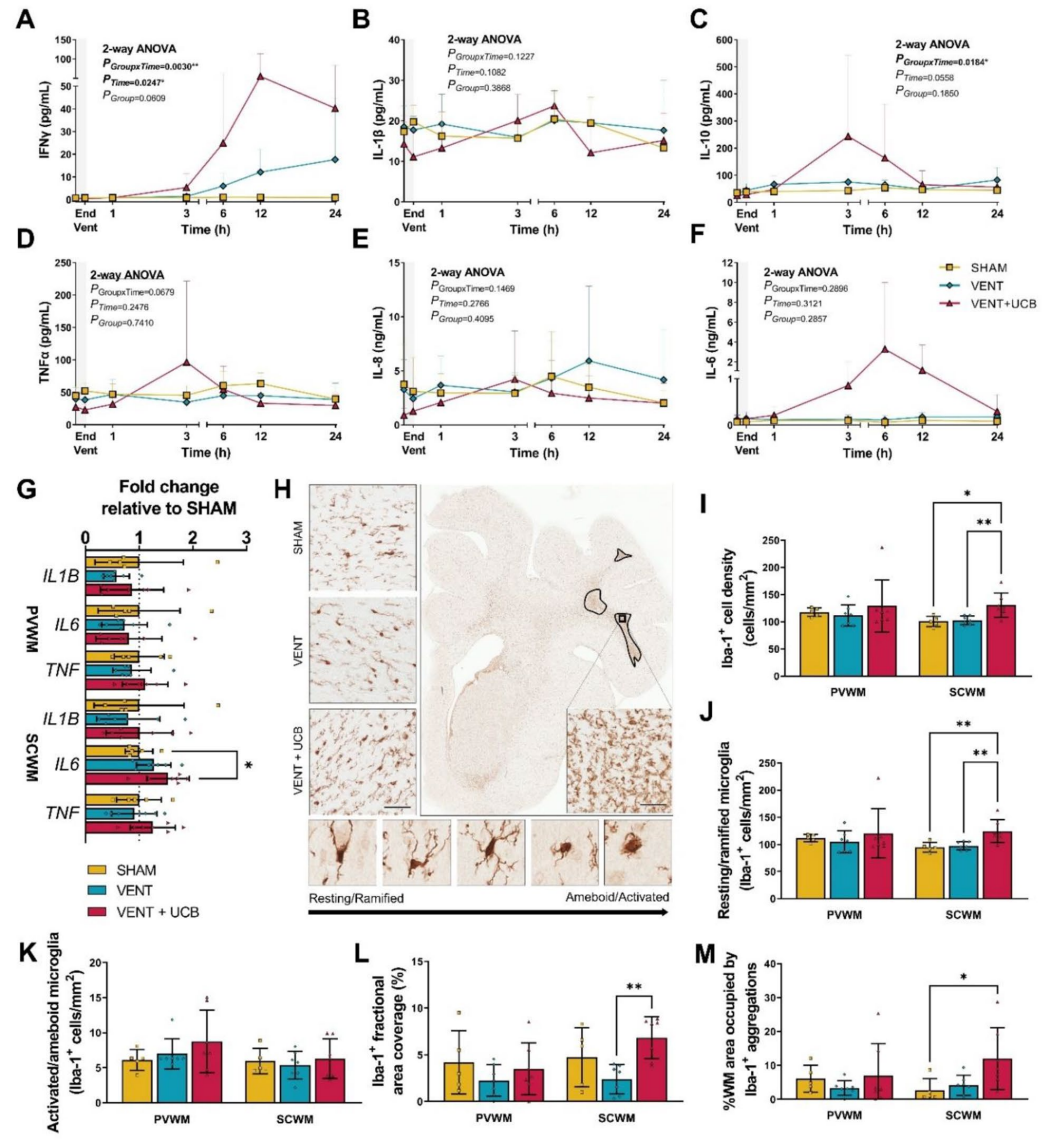
Inflammation present in the VENT + UCB group but not VENT group. Plasma cytokine levels (**A**) IFNγ, (**B**) IL-1β, (**C**) IL-10, (**D**) TNFα, (**E**) IL-8 and (**F**) IL-6 over 24 h following 15 min of brief high V_T_ ventilation (grey bar). SHAM; *n* = 5, VENT; *n* = 7 and VENT + UCB; *n* = 7. Two-Way repeated measures ANOVA. (**G**) Gene expression levels of inflammatory cytokines (interleukin 1 beta [*IL1B*], interleukin 6 [*IL6*] and tumour necrosis factor [*TNF*]) expressed as a fold change relative to SHAM. SHAM; *n* = 5, VENT; *n* = 7 and VENT + UCB; *n* = 7. One-way ANOVA. Tukey’s post-hoc comparisons; *P < 0.05. (**H**) Representative images of Iba-1 positive immunohistochemical staining in the SCWM. Insert demonstrates significant microglial aggregation. When resting, microglia display ramified morphology but as microglia are activated, they adopt an ameboid morphology. Scale bar = 50 μm. Quantitative analysis in the PVWM and SCWM of (**I**) the total number of Iba-1^+^ cells, (**J**) the number of resting/ramified microglia, (**K**) the number of ameboid microglia, (**L**) the fractional area coverage of Iba-1^+^ staining, and (**M**) the percentage of white matter occupied by aggregation. SHAM; *n* = 5, VENT; *n* = 7 and VENT + UCB; *n* = 7. One-Way ANOVA. Tukey’s post-hoc comparisons; *P < 0.05, **P < 0.01. All data are mean ± SD

Inflammation is a key driver of preterm-related white matter injury. To characterise systemic inflammation caused by brief high V_T_ ventilation and UCB cells, we measured the concentration of cytokines within arterial plasma periodically over the 24 h experimental period (Fig. 4A-F). Plasma IFNγ and IL-10 levels differed between groups over time with levels markedly increased in the VENT + UCB group compared to both VENT and SHAM (Fig. 4A,C). Plasma IL-1β, TNFα, IL-8, and IL-6 levels were not different between groups (Fig. 3B, D-F). At 24 h, mRNA expression of pro-inflammatory cytokine IL6 was increased within the subcortical white matter (SCWM) in the VENT + UCB group but not VENT only group (*P*_*vs.SHAM*_=0.037; Fig. 4G). mRNA expression of *IL1B* and *TNF* was unchanged in VENT and VENT + UCB group (Fig. 4G).

Immunohistochemical staining of Iba-1^+^ and GFAP^+^ was analysed to assess neuroinflammation/neuroglial reactivity within the cerebral white matter. Iba-1 stains microglia, the resident immune cells found within the brain. Morphological assessment delineates whether microglia are in a resting (ramified) or activated (ameboid) state [36] (Fig. 4H). Brief high V_T_ ventilation alone did not affect Iba-1^+^ staining in the PVWM or SCWM (Fig. 4I-M). The administration of UCB treatment after brief high V_T_ ventilation increased the number of total Iba-1^+^ cells compared to the VENT only group (*P* = 0.009; Fig. 4I) which was due to an increase in resting/ramified microglia (*P* = 0.008; Fig. 4J) and not the number of activated/ameboid microglia (Fig. 4K). Consistent with the increase in cell density was the increase in Iba-1^+^ fractional area coverage in the VENT + UCB group compared to VENT in the SCWM (*P* = 0.010; Fig. 4L). In addition, the VENT + UCB group had higher percentages of SCWM area covered by aggregations compared to the SHAM group (*P* = 0.027; Fig. 4M). Neither ventilation nor UCB treatment altered GFAP^+^ cell density and fractional area coverage staining (Fig.S1).

Overall, our data indicate a very minimal pro-inflammatory response in the VENT group but amplified inflammation, both systemically and within the brain, in the VENT + UCB group.

### Investigating the effect of inflammation on UCB derived mononuclear cells in vitro

**Fig. 5:**
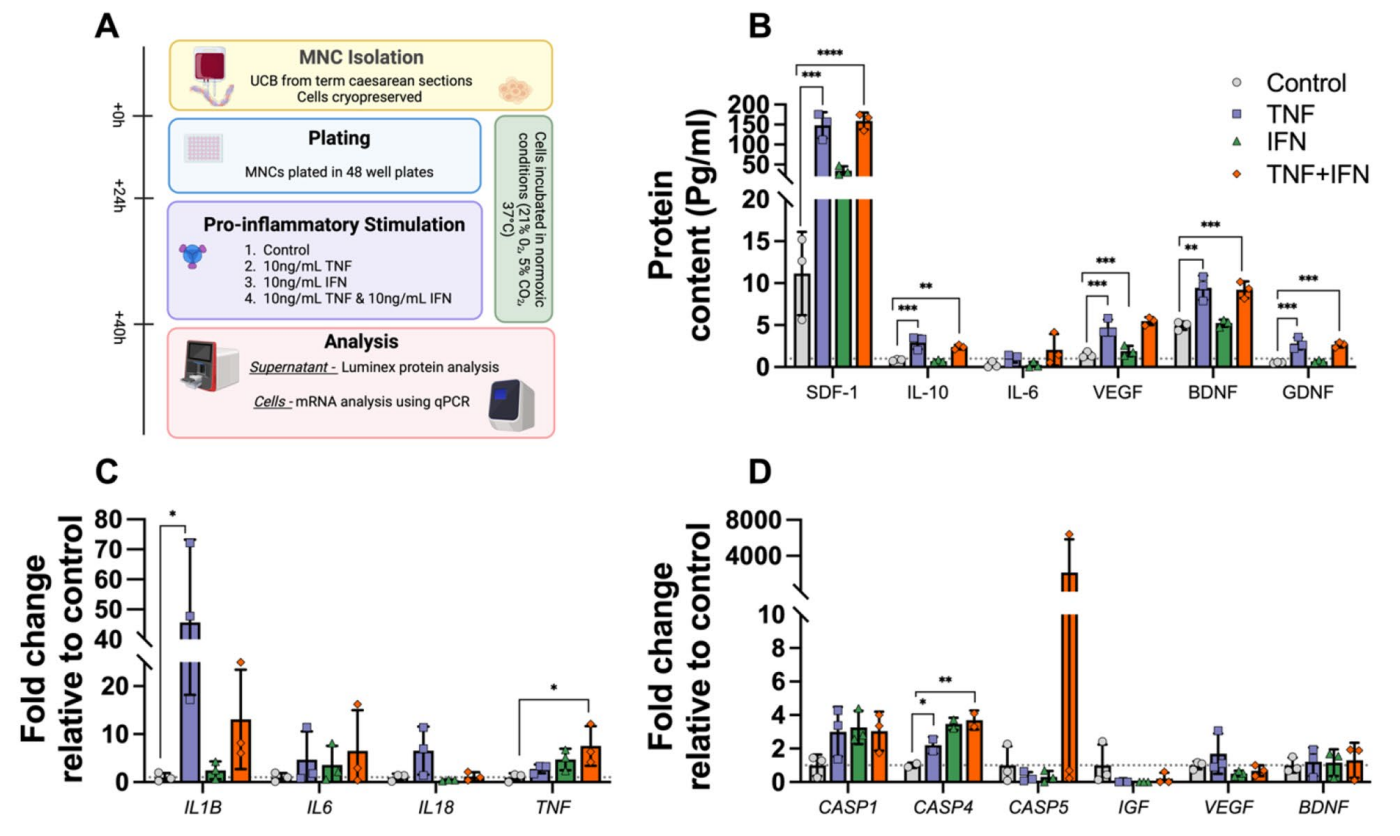
Pro-inflammatory stimulation of UCB MNCs resulted in differential expression of key cytokines, caspases and growth factors. (**A**) Schematic overview of in vitro experimental paradigm where UCB MNCs were stimulated with the pro-inflammatory factors TNFα & IFNγ for 16 h. (**B**) Conditioned media was collected to measure protein content of inflammatory-related proteins and growth factors. Cells were also harvested to measure mRNA expression levels of (**C**) inflammatory cytokines, (**D**) caspases and growth factors expressed as fold change relative to control. One-Way ANOVA, Dunnett’s post-hoc comparisons. All data are presented as mean ± SD. *P < 0.05, **P < 0.01, ***P < 0.001, ****P < 0.0001. Control (grey circles), *n* = 3; TNF (purple squares), *n* = 3; IFN (green triangles), *n* = 3; TNF + IFN (orange diamond), *n* = 3, with each performed in triplicate

Pro-inflammatory stimuli preconditioning has been shown to increase the immunomodulatory functions of other stem cells such as mesenchymal stromal cells [37], but it is unclear as to how a pro-inflammatory environment effects UCB derived MNCs. Our in vivo investigations indicate that the UCB cells may have adopted a pro-inflammatory state when administered early after ventilation-induced injury. As such, we investigated the potential mechanisms underlying the response of UCB derived MNCs in a pro-inflammatory environment in vitro; we also investigated mRNA and protein effects on individual cell types (HSCs, EPCs and monocytes) which are reported in Table S3 & S4 respectively. A pro-inflammatory environment was simulated by spiking media with the pro-inflammatory cytokines IFNγ and TNFα (10ng/mL; Fig. 5A), both known to be differentially upregulated in the brain and systemically with preterm birth and to play a role in white matter injury [38].

### Pro-inflammatory stimulation upregulated mRNA and protein expression of pro- and anti-inflammatory cytokines and chemokines

IL-1β is a major pro-inflammatory cytokine associated with preterm white matter injury [39]. mRNA expression of *IL1B* in MNCs was upregulated following TNFα stimulation (*P* = 0.015; Fig. 5C). We also found that *IL18* mRNA expression was increased following stimulation with TNFα, though this did not reach significance (*P* = 0.07; Fig. 5C). We did not observe any changes in mRNA or protein expression of IL-6 in response to pro-inflammatory stimuli (Fig. 5B and C).

TNFα is a pro-inflammatory cytokine which is known to be upregulated in the setting of brain injury, contributing to injury pathogenesis. *TNF* mRNA expression was upregulated in MNCs when stimulated with the combination of TNFα and IFNγ (*P* = 0.027; Fig. 5C). This suggests that the UCB MNCs are responding to the inflammatory environment and subsequently producing pro-inflammatory cytokines, further perpetuating the inflammatory response.

Interestingly, we also observed an increase in IL-10 expression. IL-10 is a potent anti-inflammatory cytokine predominately produced by leukocytes, a cell population found within UCB MNCs. Stimulation with TNFα and the combination of TNFα and IFNγ resulted in an increase in IL-10 expression in MNCs (*P* = 0.001 and *P* = 0.007 respectively; Fig. 5D).

In addition to inflammatory cytokines, we also investigated the effect of pro-inflammatory stimuli on the expression of key chemokines and their receptors. Stromal cell-derived factor 1 (SDF-1) is a chemokine that plays key roles in stem cell migration as well as tissue repair and regeneration. The balance between SDF-1 and its receptor chemokine receptor type 4 (CXCR4) is essential for trafficking stem cells to the site of injury, and stem cells expressing CXCR4 will migrate to sites of injury where SDF-1 expression is increased [40]. SDF-1 protein expression was significantly increased following stimulation with TNFα and the combination of TNFα and IFNγ (*P* = 0.0001 and *P* < 0.0001, respectively; Fig. 5B). We did not observe any changes in mRNA levels of *CXCR4* or C-C chemokine receptor types (*CCR1, CCR2, CCR4* & *CCR5*; Table S3).

One key role of caspases is to cleave inactive precursor pro-IL-1β and pro-IL-18 to form mature IL-1β and IL-18 which is then secreted from the cell via the NLR family pyrin domain containing 3 (NLRP3) inflammasome. We observed a significant increase in *CASP4* mRNA levels following stimulation with IFNγ and the combination of TNFα and IFNγ (*P* = 0.010 and *P* = 0.007 respectively), but no changes to *CASP1* or *CASP5* (Fig. 5D).

### Pro-inflammatory stimulation upregulated protein expression of growth factors

UCB MNCs can secrete growth factors that support and promote cellular growth and differentiation. In this study, pro-inflammatory stimulation with TNFα and combined TNFα and IFNγ resulted in increased protein expression of VEGF (*P* = 0.0006 and *P* = 0.0002, respectively), BDNF (*P* = 0.0010 and *P* = 0.0014, respectively) and GDNF (*P* = 0.0003 and *P* = 0.0004, respectively; Fig. 5B). Interestingly, pro-inflammatory stimulation did not alter growth factor mRNA expression in MNCs (Fig. 5D). The increased protein expression in the absence of mRNA expression may be due to the temporal delay in protein synthesis following transient upregulation of mRNA expression. This suggests a temporary neuroprotective role of UCB derived MNCs via a release of beneficial growth factors, that is being driven by exposure to a TNFα mediated inflammatory environment.

## Discussion

Preterm newborns often receive respiratory support after birth irrespective of their underlying pathologies, and whilst life-saving, respiratory support can contribute to brain injury [7]. We hypothesised that administration of UCB cells 1 h after 15 min of high V_T_ ventilation in the premature fetal sheep would reduce ventilation induced cerebral white matter inflammation and injury. Despite the effect of brief high V_T_ ventilation alone on cerebral white matter being relatively mild, unexpectedly, we found that early administration of UCB cells after ventilation increased diffusivity of white matter, increased cerebral inflammation, decreased BBB integrity and decreased oligodendrocyte cell population in the cerebral white matter at 24 h. To further investigate the mechanisms underlying these adverse effects, we assessed the immunomodulatory state of MNCs derived from human UCB when introduced to a pro-inflammatory environment. We discovered that UCB cells incubated in media spiked with pro-inflammatory cytokines (TNFα and IFNγ) adopted a predominantly pro-inflammatory state as characterized by upregulation of pro-inflammatory genes and production of pro- and anti-inflammatory cytokines. Nevertheless, UCB cells also upregulated specific growth factors suggesting potential neuroprotective roles of cells, even in the setting of profound inflammation. This study highlights the need to assess the timing of administration of UCB cells to preterm infants, especially in cases where infants are receiving respiratory support in periods of high systemic inflammation to avoid undesired side effects such as white matter pathology.

We previously demonstrated in the same lambs that 15 min of brief high V_T_ ventilation led to significant lung injury indicated by persisting leukocyte infiltration and structural changes [25]. Interestingly, we found that early UCB cell treatment did not reduce or exacerbate this ventilation-induced lung injury [25]. Here however, we found different responses in the preterm brain. UCB cells given during the period of peak inflammation exacerbated white matter injury when ventilation alone was benign. We suspect that returning the lambs *in utero* following ventilation causes the lungs to become liquid filled once again, reinstating the right-to-left shunting and minimal pulmonary perfusion. As the pulmonary circulation is buffered, the intravenous administration of UCB cells can only target other accessible organs such as the brain. We and others have reported extensively that UCB treatment alone does not induce systemic nor cerebral injury when administered > 12 h after the initial insult in preterm and term asphyxiated lambs [9, 26]. Therefore, the white matter injury observed in this study is likely to be due to the interaction between the systemic consequences of ventilation and the direct effect on UCB cells.

Inflammation is a key mediator of preterm brain injury [41], particularly when infants are exposed to systemic inflammation [42]. In this study, 15 min of high V_T_ ventilation induced negligible increases in systemic inflammation across the 24 h period studied, probably due to the relatively short duration of the ventilation intervention. However, the intravenous administration of UCB cells following ventilation increased systemic cytokines which persisted during the 24 h. We theorise that the transient high V_T_ ventilation created an acute inflammatory environment and the introduction of the UCB cells to this environment inappropriately triggered the cells’ innate pro-inflammatory response mechanisms to cause downstream neuropathological consequences. A key mechanism by which the brief high V_T_ ventilation may have increased the susceptibility of the white matter to further damage is alterations to the BBB. Ventilation induces BBB permeability within minutes in preterm lambs [43], with evidence of brain diffusion and metabolic disturbances by 1 h following high V_T_ ventilation [27]. Indeed, we observed evidence of altered endothelial integrity within the white matter as indicated by a downregulation of tight junction proteins, but did not observe histological evidence of increased BBB permeability in VENT lambs. The lack of disturbed BBB integrity at 24 h likely reflects a recovery from the transient permeability induced by ventilation. Complete BBB permeability recovery has been shown to occur at 24 h after 15 min of cerebral ischemia in adult rats [44]; and thus the 24 h of normalised cerebral reperfusion in the absence of continued ventilation likely explains the intact BBB compared to the other preterm lamb studies reported BBB permeability following ventilation [27, 43]. A disturbance to the BBB integrity may have rendered the immature brain temporally vulnerable to infiltration of systemic inflammatory cytokines, leukocytes and trophic factors that promoted localized cerebral inflammation from the UCB cells.

Pro-inflammatory preconditioning of cell therapies, such as mesenchymal stromal cells (MSCs), has been shown to increase their immunosuppressive and reparative capacity [37]. Little research has been conducted to assess the effect of pro-inflammatory stimulation on UCB MNCs and the individual cells within this population. UCB MNCs are comprised of many stem and progenitor cells, including MSCs, hematopoietic stem cells (HSC), endothelial progenitor cells (EPC) and monocytes, and thus it is important to understand how each of these cell types respond to inflammatory stimulation individually, and together. Here, we demonstrate that MNCs derived from human UCB increased gene expression of pro-inflammatory cytokines and chemokines, with corresponding increases in protein when stimulated with pro-inflammatory stimuli in vitro. This reflects the increase of pro-inflammatory cytokines seen in vivo, further confirming that when placed into a suboptimal environment UCB cells will mount an inflammatory response. Adding to the complexity, MNCs also upregulated anti-inflammatory cytokine and growth factor release following pro-inflammatory stimulation suggesting potential reparative responses. Specifically, there was an increase in IL-10 expression, which is consistent with the in vivo study where we observed a transient increase in IL-10 protein expression following UCB administration. There was also an increase in expression of the growth factors VEGF, GDNF and BDNF, which highlights a neuroprotective role of UCB cells in the setting of inflammation. These growth factors are known to modulate injury in response to neonatal hypoxia ischemia via anti-inflammatory, anti-apoptotic and pro-angiogenic mechanisms [45–47]. UCB is a heterogeneous population of cells and thus we further investigated which cell populations may be responsible for driving inflammation. We individually measured gene and protein expression of HSCs, EPCs and monocytes, when exposed to a pro-inflammatory environment, however we were unable to extrapolate any discernable pattern to identify which predominate cell population was driving the responses observed within the total MNC population (Table S3 & S4).

On the basis of our findings in preterm sheep, we suggest that caution should be taken in future studies if UCB cells are to be used in preterm infants as an early therapeutic intervention strategy for preterm brain injury. The growing interest in, feasibility and safety profile of UCB cells for preterm infants encourages their early administration to optimise neuroprotection [4, 9, 48, 49], however in these studies “early” treatment is still > 12 h post initial insult. Notwithstanding the other morbidities that are likely to occur in preterm infants, the majority of preterm infants require respiratory support for varying periods of time. If UCB cells are to be administered as a treatment strategy for morbidities such as hypoxic-ischemic encephalopathy or intraventricular haemorrhage, the timing of administration in relation to the pathogenesis of inflammatory processes, especially following ventilatory interventions, must be considered. This is critical as up to 96% of infants < 32 weeks GA receive respiratory support at birth [50] which can occur for a duration of days to weeks [51]. The neuroinflammatory processes following prolonged ventilation most likely differs to a brief high V_T_ ventilation method; for example, we have shown persisting increased levels of systemic cytokines during 24 h of *in utero* ventilation with V_T_ of 3 mL/kg in fetal sheep with evidence of heightened microglial and astrocytic responses within the brainstem [52]. Whether UCB cells demonstrate a similar pro-inflammatory state in cases of prolonged ventilation or ventilation with other co-morbidities requires further interrogation.

The use of UCB for ventilation-induced brain and lung injury should not be dismissed however, as the timing for administration may be key to its efficacy. Both preclinical and clinical studies utilising UCB cell therapy as a treatment for perinatal-related complications have demonstrated particular efficacy when administration occurs after 6–24 h after birth/injury [6, 9]. This timing can even be delayed to 3–10 days after preterm birth, with UCB cells and UCB-derived cells reducing intraventricular haemorrhage rates and bronchopulmonary dysplasia (BPD) severity [53, 54]. It is also important to note that in our previous study by Li et al., demonstrating beneficial effects of allogeneic UCB cells, UCB cells preparation was the same but cells were administered 12 h after the insult [9]. The optimal therapeutic window for administration may therefore be *after* peak inflammatory mechanisms have occurred whereby UCB cells would play a more restorative role rather than as a preventative therapeutic strategy.

A major limitation of our study is the absence of measurable white matter injury or systemic inflammation following exposure to mechanical ventilation that we have shown previously [7, 17, 18, 55]. This suggests a subclinical manifestation of the injury, creating a subtle pro-inflammatory environment that was adequate to induce a pro-inflammatory response of UCB cells. Future studies should consider a more injurious model to fully explore the effects of UCB cells in the setting of established brain inflammation/injury. A further limitation was our inability to ascertain the exact mechanisms by which the UCB cells exacerbated white matter injury following acute mechanical ventilation. Due to the experimental paradigm, we were unable to investigate how the UCB cellular constituents reacted to the cytokines at a temporal and spatial level, future investigations using metabolomics and proteomics platforms may reveal these exact mechanisms. We did not label the UCB cells in this study and could not trace if they entered the brain parenchyma, though we know that UCB cells are present in low numbers in the brain after intravenous administration in preterm [9] and near-term [26] lambs and that their effects are predominantly due to trophic actions. We have identified multiple mechanisms by which UCB cells can adopt an injurious state – whether these mechanisms are unique to ventilation-induced injury or are also involved in other perinatal pathologies needs further investigation. In this study we have demonstrated an increase in neuroinflammation and a decrease in white matter integrity at 24 h post-injury, however due to the short-term nature of this study we do not know if these changes will persist or if they are transient and thus may resolve over time. To fully ascertain this, future studies should include later timepoints to determine how injury progresses after ventilation and UCB administration. Multiple iterations of ventilation-induced brain injury need to be assessed to ascertain the exact interaction between ventilation and UCB cells; e.g., prolonged ventilation and UCB administration during ventilation or after ventilation cessation.

## Conclusion

Overall, we demonstrate that a single systemic dose of allogeneic UCB cells 1 h after acute high V_T_ ventilation in preterm lambs induced damage to the integrity of white matter tracts, compromised vascular integrity and increased neuroinflammation at 24 h. Our results suggest that brief ventilation sensitises the preterm brain to injury, and the early introduction of UCB cells into a vulnerable or pro-inflammatory microenvironment may exacerbate inflammatory responses that can lead to white matter injury. Future clinical studies using UCB cells should consider how ventilation, and other common interventions at birth, may alter the inflammatory environment, and thus how UCB cells will subsequently respond to this environment. As such, caution for early administration in preterm infants needs to be observed.

### Electronic supplementary material

Below is the link to the electronic supplementary material.

**Supplementary Material 1: Fig. S1:** No changes to GFAP immunoreactivity in the white matter

**Supplementary Material 2: Table S1:** Details of genes investigated using RT-qPCR by Fluidigm on frozen tissue

**Supplementary Material 3: Table S2:** Primer sequences for in vitro gene expression analysis

**Supplementary Material 4: Table S3:** mRNA expression in in vitro study

**Supplementary Material 5: Table S4:** Protein expression in in vitro study

**Supplementary Material 6: Data file S1:** ARRIVE Checklist for animal research

## Data Availability

Data is provided within the manuscript or supplementary information files. The datasets used during the current study are available from the corresponding author upon reasonable request.

## Abbreviations

°C: Degrees Celsius
AD: Axial diffusivity
ANOVA: Analysis of variance
AU: Arbitrary units
BBB: Blood-brain-barrier
BDNF: Brain-derived neurotrophic factor
CASP-: Caspase-
Cat#: Catalog number
CCR-: C-C chemokine receptor type-
cDNA: Complementary Deoxyribonucleic acid
CLDN1: Claudin 1
CXCR-: Chemokine receptor type-
dGA: Days gestational age
DMEM: Dulbecco’s Modified Eagle Medium
DMSO: Dimethylsulfoxide
DTI: Diffusion tensor imaging
DWI: Diffusion weighted imaging
EDTA: Ethylenediaminetetraacetic acid
FA: Fractional anisotropy
FBS: Fetal bovine serum
FiO2: Fraction of inspired oxygen
*g*: G-force
g: Grams
GDNF: Glial cell line-derived neurotrophic factor
GFAP: Glial fibrillary acidic protein
h: Hour
Iba-1: Ionised calcium binding adapter molecule-1
IFN: Interferon
IGF: Insulin-like growth factor
IgG: Immunoglobulin G
IL: Interleukin
kg: Kilogram
MBP: Myelin basic protein
MD: Mean diffusivity
mg: Micrograms
min: Minutes
mL: Milliliter
mm: Millimeters
mm: Milometers
MNCs: Mononuclear cells
MRI: Magnetic resonance imaging
mRNA: Messenger ribonucleic acid
*n*: Number
ng: Nanograms
NLRP3: NLR family pyrin domain containing 3
OCLN: Occludin
Olig2: Oligodendrocyte transcription factor-2
*P*: *p*-value
P53: Tumor protein 53
PaCO_2_: Partial pressure of carbon dioxide
PaO_2_: Partial pressure of oxygen
PBS: Phosphate-buffered saline
PCR: Polymerase Chain Reaction
PEEP: Peak end-expiratory pressure
pg: Picogram
PIP: Peak inspiratory pressure
PVWM: Periventricular white matter
RD: Radial diffusivity
RNA: Ribonucleic acid
ROI: Region of interests
Rpm: Rotations per minute
RT-PCR: Reverse transcription polymerase chain reaction
SaO_2_: Oxygen saturation
SCWM: Subcortical white matter
SD: Standard deviation
SDF-1: Stromal cell-derived factor 1
TCEP: Tris(2-carboxyethyl)phosphine
TNF: Tumor necrosis factor
TUNEL: Terminal deoxynucleotidyl transferase dUTP nick end labelling
UCB: Umbilical cord blood
VEGF: Vascular endothelial growth factor
VENT: Ventilation
V_T_: Tidal volume
µL: Microliter
µm: Micrometre
