# Supplementary figures and images for: Early administration of umbilical cord blood cells following brief high tidal volume ventilation in preterm sheep: a cautionary tale
Source: J Neuroinflammation. 2024 May 8;21:121. doi: 10.1186/s12974-024-03053-3 (PMC11077893; doi:10.1186/s12974-024-03053-3)

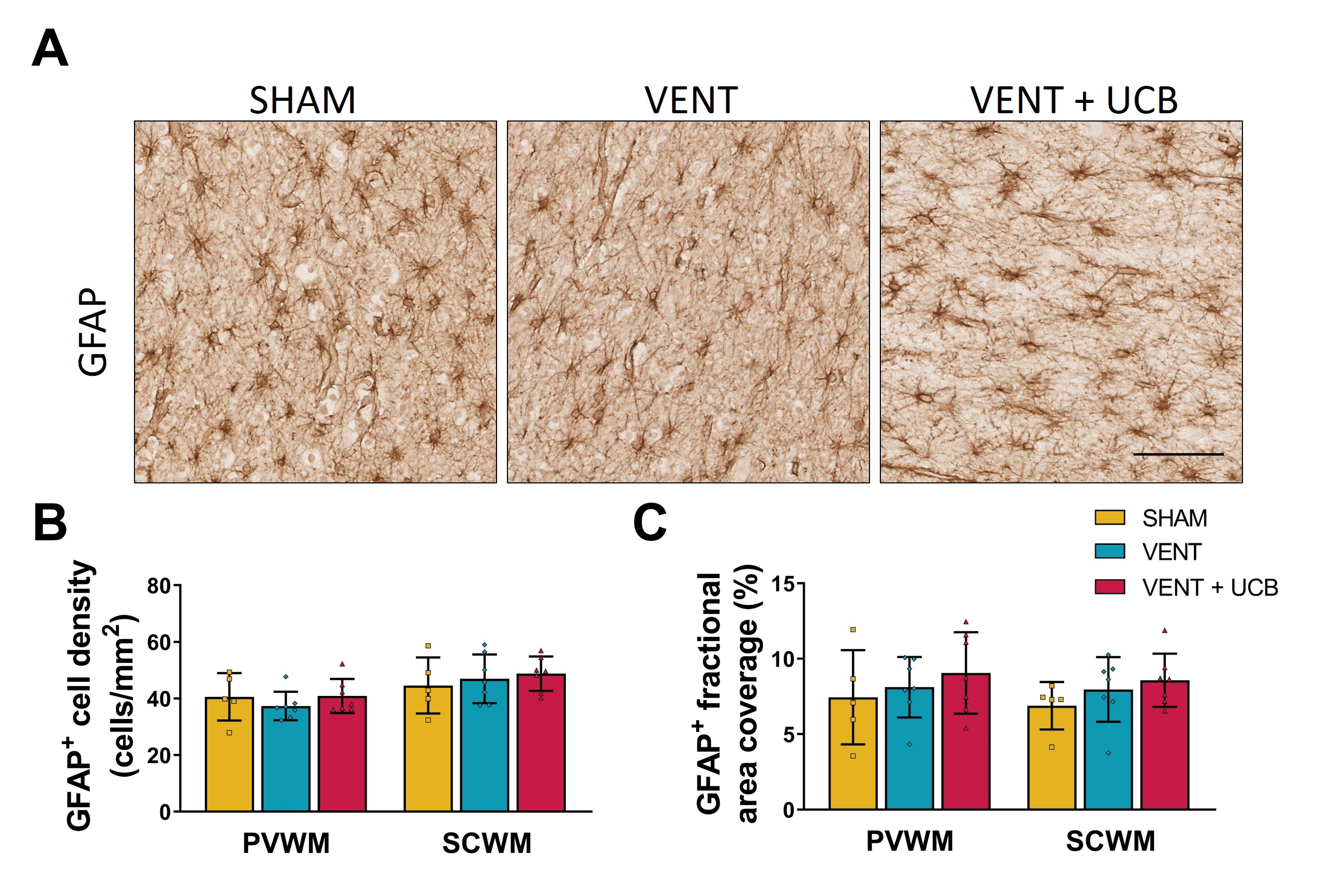

Supplement: Supplementary file 1 — Supplementary Material 1: Fig. S1: No changes to GFAP immunoreactivity in the white matter [file 12974_2024_3053_MOESM1_ESM.jpg]
