# Supplementary material for: Early administration of umbilical cord blood cells following brief high tidal volume ventilation in preterm sheep: a cautionary tale
Source: J Neuroinflammation. 2024 May 8;21:121. doi: 10.1186/s12974-024-03053-3 (PMC11077893; doi:10.1186/s12974-024-03053-3)
Supplement: Supplementary file 2 — Supplementary Material 2: Table S1: Details of genes investigated using RT-qPCR by Fluidigm on frozen tissue [file 12974_2024_3053_MOESM2_ESM.docx]

**Table S1.** **Details of genes investigated using RT-qPCR by Fluidigm on frozen tissue.** The TaqMan assay ID for each probe is provided; target-specific sequences of TaqMan assays are not available due to non-disclosure policies.

| **Cell pathway** | **Gene name** | **Gene symbol** | **TaqMan Assay ID** |
| --- | --- | --- | --- |
| Reference gene | Ribosomal protein S18 | *RPS18* | Oa04906333_g1 |
| Inflammatory cytokines | Interleukin 1 beta | *IL1B* | Oa04656322_m1 |
|  | Interleukin 6 | *IL6* | Oa04656315_m1 |
|  | Tumour necrosis factor alpha | *TNF* | Oa04655425_g1 |
| Markers of cell death | Tumor protein p53 | *P53* | Oa03223218_g1 |
|  | Caspase 3 | *CASP3* | Oa04817361_m1 |
| Tight junction proteins | Occludin | *OCLN* | Oa04728970_m1 |
|  | Claudin 1 | *CLDN1* | Oa03217991_m1 |
