# Supplementary material for: Early administration of umbilical cord blood cells following brief high tidal volume ventilation in preterm sheep: a cautionary tale
Source: J Neuroinflammation. 2024 May 8;21:121. doi: 10.1186/s12974-024-03053-3 (PMC11077893; doi:10.1186/s12974-024-03053-3)
Supplement: Supplementary file 3 — Supplementary Material 3: Table S2: Primer sequences for in vitro gene expression analysis [file 12974_2024_3053_MOESM3_ESM.docx]

**Table S2. Primer sequences for *in vitro* gene expression analysis.**

| Gene Primer | |  | Sequence |
| --- | --- | --- | --- |
| *CXCR4* | Forward | | CCAGTAGCCACCGCATCT |
|  | Reverse | | AACAGGGTTCCTTCATGGAGTC |
| *CCR1* | Forward | | AGAGCCAATCAGTAGCCAGC |
|  | Reverse | | CCAGGATTCCAAGAGCCCAA |
| *CCR2* | Forward | | TTGGGGAGAAGTTCAGAAGCC |
|  | Reverse | | TTTGGAGTGGGGCAATCCTAC |
| *CCR4* | Forward | | AAAGCAAGCTGCTTCTGGTTG |
|  | Reverse | | GGTGTCTGCTATATCCGTGGG |
| *CCR5* | Forward | | TCTATGAGGCAACCACAGGC |
|  | Reverse | | ATGGGACCCCTTTGCTCAAT |
| *MCP1* | Forward | | AACTGAAGCTCGCACTCTCG |
|  | Reverse | | GGGGCATTGATTGCATCTGG |
| *CASP1* | Forward | | GGAAACAAAAGTCGGCAGAG |
|  | Reverse | | ACGCTGTACCCCAGATTTTG |
| *IL1B* | Forward | | GCCATGGCAGAAGTACCTGA |
|  | Reverse | | GTCCTGGAAGGAGCACTTCAT |
| *CASP5* | Forward | | TGTTAGCTATGGCTGCTGTG |
|  | Reverse | | TGTCCAGCCACGTTGTTCTT |
| *CASP4* | Forward | | AAGAGAAGCAACGTATGGCAGGAC |
|  | Reverse | | GGACAAAGCTTGAGGGCATCTGTA |
| *TNF* | Forward | | GCCCATGTTGTAGCAAACCC |
|  | Reverse | | GGAGGTTGACCTTGGTCTGG |
| *IL18* | Forward | | TGCAGTCTACACAGCTTCGG |
|  | Reverse | | CCAGGTTTTCATCATCTTCAGC |
| *IL6* | Forward | | TCAATATTAGAGTCTCAACCCCCAA |
|  | Reverse | | GAAGGCGCTTGTGGAGAAGG |
| *GDNF* | Forward | | AGTCAAGAGAGGGTTTTCGGG |
|  | Reverse | | GACAGCCACGACATCCCATA |
| *IGF* | Forward | | ATCAGCAGTCTTCCAACCCAAT |
|  | Reverse | | GCCAGGTAGAAGAGATGCGA |
| *VEGF* | Forward | | CTTTCTGCTGTCTTGGGTGC |
|  | Reverse | | ACTTGGCATGGTGGAGGTAG |
| *BDNF* | Forward | | CTGCTTTCTCCCTACAGTTCCA |
|  | Reverse | | CCACCTTGTCCTCGGATGTT |
| *β-actin^*^* | Forward | | GGGCATGGGTCAGAAGGATT |
|  | Reverse | | AGTTGGTGACGATGCCGTG |
