# Supplementary material for: Early administration of umbilical cord blood cells following brief high tidal volume ventilation in preterm sheep: a cautionary tale
Source: J Neuroinflammation. 2024 May 8;21:121. doi: 10.1186/s12974-024-03053-3 (PMC11077893; doi:10.1186/s12974-024-03053-3)
Supplement: Supplementary file 4 — Supplementary Material 4: Table S3: mRNA expression in in vitro study [file 12974_2024_3053_MOESM4_ESM.docx]

**Table S3.** **mRNA expression of in vitro study.** Average mRNA expression levels of all genes analysed in this study in cultured MNCs and individual cell types: monocytes, EPCs and HSCs. Values expressed as fold change from control. **P*<0.05, #*P*<0.01, $*P*<0.001, ^*P*<0.0001

|  | MNC | | | Monocytes | | | EPCs | | | HSCs | | |
| --- | --- | --- | --- | --- | --- | --- | --- | --- | --- | --- | --- | --- |
|  | **TNF** | **IFN** | **TNF+IFN** | **TNF** | **IFN** | **TNF+IFN** | **TNF** | **IFN** | **TNF+IFN** | **TNF** | **IFN** | **TNF+IFN** |
| CXCR4 | 2.05 | 0.73 | 1.78 | 0.81 | 0.20* | 0.27 | 2.24 | 3.77 | 0.99 | 1.03 | 0.96 | 0.36* |
| CCR1 | 1.98 | 1.49 | 1.08 | 1.09 | 0.85 | 1.63 | 2.78 | 5.39 | 5.36 | 0.52^#^ | 0.97 | 0.87 |
| CCR2 | 0.09 | 0.01 | 0.04 | 0.28^#^ | 0.22^#^ | 0.07^#^ | 2.60 | 0.72 | 0.37 | 0.51 | 0.89 | 0.18 |
| CCR4 | 1.50 | 0.92 | 0.74 | 1.00 | 0.28 | 0.22 | 1.00 | 4.32 | 5.27 | 2.43* | 1.31 | 1.31 |
| CCR5 | 3.00 | 1.84 | 3.17 | 1.57 | 1.28 | 2.81* | 2.11 | 5.37 | 7.05 | 0.65 | 0.99 | 1.25 |
| MCP1 | 7.99 | 3.59 | 4.75 | 1.47 | 1.20 | 2.62* | 16.90 | 2.93 | 11.85 | 3.49 | 0.37 | 1.32 |
| IL1B | 45.69* | 2.46 | 13.06 | 8.22^#^ | 0.67 | 7.74^#^ | 0.89 | 0.72 | 0.72 | 1.82* | 0.71 | 0.85 |
| IL6 | 4.68 | 3.60 | 6.50 | 0.23 | 0.15 | 0.08 | 4.42 | 22.70 | 35.56 | 0.38 | 2.40 | 1.01 |
| IL18 | 6.56 | 0.39 | 1.16 | 1.33 | 0.06 | 0.09 | 4.34 | 5.00 | 7.98 | 0.45 | 2.28 | 0.51 |
| TNF | 2.77 | 4.72 | 7.54* | 2.25 | 7.77 | 10.78 | 0.87 | 2.13 | 213.55 | 2.09 | 2.80 | 5.44* |
| CASP1 | 3.01 | 3.26 | 3.03 | 0.93 | 3.06^#^ | 4.14^$^ | 0.98 | 2.71 | 1.89 | 0.46 | 3.40^#^ | 1.54 |
| CASP4 | 1.47 | 2.31* | 2.47^#^ | 0.29 | 0.86 | 0.91 | 0.65 | 0.97 | 19.96 | 0.42 | 2.22* | 1.10 |
| CASP5 | 0.30 | 0.31 | 2,138.00 | 0.41 | 3.09 | 0.29 | 0.03 | 60,244.65 | 2.61 | 1.68 | 4.09 | 2.75 |
| IGF | 0.02 | 0.01 | 0.21 | 1.70 | 0.17 | 0.52 | 0.23 | 1.16 | 0.59 | 19.91 | 1.99 | 2.10 |
| VEGF | 1.69 | 0.51 | 0.68 | 11.74 | 0.83 | 2.07 | 4.35 | 10.16 | 4.83 | 68.20 | 2.76 | 1.09 |
| BDNF | 1.21 | 1.16 | 1.30 | 2.01 | 0.81 | 4.15 | 0.84 | 4.13 | 201.34 | 12.31 | 2.21 | 0.45 |
|  | | | | | | | | | | | | |
