# Supplementary material for: Early administration of umbilical cord blood cells following brief high tidal volume ventilation in preterm sheep: a cautionary tale
Source: J Neuroinflammation. 2024 May 8;21:121. doi: 10.1186/s12974-024-03053-3 (PMC11077893; doi:10.1186/s12974-024-03053-3)
Supplement: Supplementary file 5 — Supplementary Material 5: Table S4: Protein expression in in vitro study [file 12974_2024_3053_MOESM5_ESM.docx]

**Table S4. Protein expression in in vitro study.** Average expression of all proteins assessed in this study measured in conditioned media from cultured MNCs and individual cell types: monocytes, EPCs and HSCs. Values are average protein content (pg/mL). **P*<0.05, #*P*<0.01, $*P*<0.001, ^*P*<0.0001 compared to control.

|  | MNC | | | | Monocytes | | | | EPCs | | | | |  | | HSCs | |  | |
| --- | --- | --- | --- | --- | --- | --- | --- | --- | --- | --- | --- | --- | --- | --- | --- | --- | --- | --- | --- |
|  | **Control** | **TNF** | **IFN** | **TNF+IFN** | **Control** | **TNF** | **IFN** | **TNF+IFN** | | **Control** | **TNF** | **IFN** | **TNF+IFN** | **Control** | **TNF** | | **IFN** | | **TNF+IFN** |
| Angpt1 | 1690.3 | 1548.6 | 1512.9 | 1762.5 | 1346.1 | 1329.8 | 1308.9 | 1275.4 | | 1716.9 | 1749.4 | 1628.8 | 1673.1 | 1889.9 | 1910.9 | | 1868.2 | | 1880.8 |
| SDF-1 | 11.1 | 148.2^$^ | 44.6 | 158.9^^^ | 120.6 | 206.8^$^ | 142.3 | 207.4^$^ | | 91.2 | 198.5* | 120.4 | 213.5* | 24.2 | 155.8^^^ | | 65.3* | | 156.3^^^ |
| IFN | 8.6 | 34.8 | 4545.0^^^ | 4813.5^^^ | 24.8 | 50.0 | 2984.7* | 4823.9^#^ | | 19.7 | 46.5 | 3164.9* | 3508.9^#^ | 7.8 | 31.5 | | 2796.6^$^ | | 3537.6^$^ |
| TNF | 0.2 | 14812.3* | 4.1 | 8466.3 | 2.3 | 15606.5 | 2.4 | 10827.8 | | 2.5 | 12268.2^^^ | 2.9 | 11876.4^^^ | 0.5 | 11052.5^^^ | | 1.4 | | 9073.4^^^ |
| IL-6 | 0.3 | 1.0 | 0.3 | 2.1 | 3.3 | 4.4 | 4.0 | 2.5 | | 13.2 | 92.8 | 4.5 | 10.4 | 5.9 | 18.7 | | 5.1 | | 6.0 |
| IL-10 | 0.8 | 2.9^$^ | 0.7 | 2.4^#^ | 5.2 | 6.7^#^ | 5.5 | 6.7^#^ | | 3.9 | 5.5 | 3.7 | 6.3 | 0.7 | 2.6^^^ | | 1.1^#^ | | 2.7^^^ |
| VEGF | 1.4 | 4.7^$^ | 1.9 | 5.5^$^ | 6.3 | 8.6^#^ | 6.5 | 9.0^#^ | | 4.7 | 7.5 | 4.8 | 8.5 | 1.0 | 3.8^^^ | | 1.2 | | 4.2^^^ |
| BDNF | 4.9 | 9.5^$^ | 5.2 | 9.2^#^ | 4.0 | 8.4^$^ | 4.2 | 8.3^$^ | | 4.0 | 8.7^#^ | 4.4 | 9.0^#^ | 3.1 | 7.3^^^ | | 3.3 | | 7.5^^^ |
| GDNF | 0.5 | 2.8^$^ | 0.7 | 2.7^$^ | 3.8 | 6.3^#^ | 4.2 | 6.2^#^ | | 2.8 | 5.4 | 3.1 | 6.1 | 0.6 | 2.7^^^ | | 0.6 | | 2.8^^^ |
|  | | | | | | | | | | | | | | | | | | | |
